# Dietary fat intake and risk of ovarian cancer in the NIH-AARP Diet and Health Study

**DOI:** 10.1038/bjc.2011.572

**Published:** 2012-01-05

**Authors:** M M Blank, N Wentzensen, M A Murphy, A Hollenbeck, Y Park

**Affiliations:** 1 Division of Cancer Epidemiology and Genetics, National Cancer Institute, National Institutes of Health, 6120 Executive Boulevard, Rockville, MD 20852, USA; 2 Department of Epidemiology, Harvard School of Public Health, Boston, MA 02115, USA; 3 AARP, 601 E Street NW, Washington, DC 20049, USA

**Keywords:** dietary fat, ovarian cancer, cohort studies

## Abstract

**Background:**

Fat intake has been postulated to increase risk of ovarian cancer, but previous studies have reported inconsistent results.

**Methods:**

The NIH-AARP Diet and Health Study, a large prospective cohort, assessed diet using a food frequency questionnaire at baseline in 1995–1996. During an average of 9 years of follow-up, 695 ovarian cancer cases were ascertained through the state cancer registry database. The relative risks (RRs) and 95% confidence interval (CI) were estimated using a Cox proportional hazard model.

**Results:**

Women in the highest *vs* the lowest quintile of total fat intake had a 28% increased risk of ovarian cancer (RR_Q5 *vs* Q1_=1.28, 95% CI: 1.01–1.63). Fat intake from animal sources (RR_Q5 *vs* Q1_=1.30; 95% CI: 1.02–1.66), but not from plant sources, was positively associated with ovarian cancer risk. Saturated and monounsaturated fat intakes were not related to risk of ovarian cancer, but polyunsaturated fat intake showed a weak positive association. The association between total fat intake and ovarian cancer was stronger in women who were nulliparous or never used oral contraceptives.

**Conclusion:**

Fat intake, especially from animal sources, was related to an increased risk of ovarian cancer. The association may be modified by parity and oral contraceptive use, which warrants further investigation.

Ovarian cancer, which is often diagnosed at an advanced stage, is the eighth most common cancer and the fifth leading cause of cancer death in women in the United States (Kohler *et al*, 2011). Although the causes of ovarian cancer have been hypothesised as environmental factors, few modifiable risk factors have been identified. Among known risk factors, oral contraceptive use is associated with a lower risk of ovarian cancer, while menopausal hormone therapy (MHT) use corresponds to an increased risk (Schottenfeld and Fraumeni, 2006). Both oral contraceptive and MHT uses are speculated to have a role in ovarian cancer aetiology partially by affecting hormones such as gonadotropins, androgens, progesterone, and oestrogens, which may promote ovarian carcinogenesis (Lukanova and Kaaks, 2005). Another factor that is postulated to increase risk of ovarian cancer by altering hormone levels, particularly oestrogen levels, is high-dietary fat intake (Cramer and Welch, 1983; Wu *et al*, 1999). Nevertheless, observational studies have reported inconsistent results and a recent expert panel report could not make a judgment on the association between fat intake and risk of ovarian cancer due to limited evidence (World Cancer Research Fund AIfCR, 2007). An early meta-analysis of seven case–control studies and one prospective cohort study (Huncharek and Kupelnick, 2001) summarised that total fat intake was significantly related to risk of ovarian cancer (relative risk (RR)=1.24, 95% confidence interval (CI): 1.07–1.43 comparing the highest *vs* the lowest category). However, a recent pooled analysis of 12 cohort studies (Genkinger *et al*, 2006) found no association between total fat intake and ovarian cancer risk, but weak positive associations with saturated fat intake and fat intake from animal foods. Comparing the highest with the lowest quartile of intake, the RR was 1.14 (95% CI: 0.97–1.34) for saturated fat intake and 1.15 (95% CI: 0.99–1.33) for fat intake from animal foods. Given that the range of fat intake across studies was relatively narrow, the pooled analysis may have limited power to detect an association, if it exists. Therefore, we examined the association between fat intake and risk of ovarian cancer in a large prospective cohort study that included 695 ovarian cancer cases and had a wide range of fat intake. We further investigated sources and types of fat and histological subtypes of ovarian cancer. In addition, we tested whether the association between fat intake and ovarian cancer differed by oral contraceptive use, parity, MHT use, and body mass index (BMI).

## Materials and methods

### Study population

The NIH-AARP Diet and Health Study was initiated in 1995–1996 when a self-administered questionnaire was sent out only once to 3.5 million men and women, aged 50–71 years, who were members of the AARP (formerly known as the American Association of Retired Persons) and resided in one of six states (California, Florida, Louisiana, New Jersey, North Carolina, and Pennsylvania) or two metropolitan areas (Atlanta, Georgia or Detroit, Michigan). The response to the one-time mailing was 17.6%. Details of the study have been described previously (Schatzkin *et al*, 2001).

Of the 567 169 participants who returned the baseline questionnaire, we excluded individuals who returned duplicate questionnaires (*n*=179), requested to be withdrawn (*n*=7), had moved out of the study area or died before baseline (*n*=582), responded as proxies for the intended respondents (*n*=15 760), were male (*n*=325 174), had any prevalent cancer other than non-melanoma skin cancer at baseline (*n*=23 953), had end-stage renal disease (*n*=371), or had undergone bilateral oophorectomy (*n*=48 145). In addition, we excluded individuals who reported extreme intakes (greater than twice the interquartile ranges above the 75th percentile or below the 25th percentile of sex-specific log-transformed intake) of total energy (*n*=1300) and total fat (*n*=146). After these exclusions, the analytical cohort consisted of 151 552 women. The study was approved by the Special Studies Institutional Review Board of the National Cancer Institute (NCI).

### Diet and risk-factor assessment

We assessed dietary intake with a 124-item food frequency questionnaire that was based on the earlier version of the NCI Diet History Questionnaire. We assessed frequency of food and beverage consumption for the previous 12 months using 10 pre-defined categories of intake ranging from ‘never’ to ‘6+ times per day’ for beverages, and ‘never’ to ‘2+ times per day’ for solid foods. Portion sizes and nutrient intakes were estimated from the 1994–1996 US Department of Agriculture's Continuing Survey of Food Intakes by Individuals. The food frequency questionnaire included 21 questions about consumption of foods that were low fat and those that contained added fats or creamers used in food preparation. In addition to total fat, we also estimated fat intake by sources of fat, including fat from animal sources (e.g., meat, egg, dairy, butter, etc.) and fat from plant sources (e.g., vegetable oil, margarine, etc.), and by types of fat, including saturated, monounsaturated, polyunsaturated and trans fat.

The food frequency questionnaire was validated in a calibration study (*n*=1953) using two nonconsecutive 24-h dietary recalls as a reference method (Thompson *et al*, 2008). The energy-adjusted Pearson correlation coefficients for the food frequency questionnaire and the 24-h dietary recalls in women were 0.62 for total fat, 0.69 for saturated fat, 0.62 for monounsaturated fat, and 0.56 for polyunsaturated fat.

The baseline questionnaire also collected information about non-diet risk factors, including demographic characteristics (e.g., race/ethnicity and level of education), height, body weight, medical history (e.g., heart disease, stroke, diabetes), family history of cancer, smoking status, time since quitted smoking, number of cigarettes per day, oral contraceptive use, MHT use, and reproductive history (e.g., parity, age at first birth, age at menarche, age at menopause). A subsequent questionnaire was mailed to people who did not have self-reported prevalent cancer at baseline within 6 months from the time the baseline questionnaire (response rate=62%). It asked for more detailed family history of cancer including ovarian cancer and medical history (e.g., hypertension, medication use).

### Cohort follow-up

Study participants were followed by annual matching of the cohort database with the National Change of Address database maintained by the US Postal Service and through processing of undeliverable mail, other address update services and direct responses from participants. Vital status was ascertained by linkage to the US Social Security Administration Death Master File with verification in the National Death Index Plus. The loss-to-follow-up is less than 5%.

### Cancer ascertainment

We identified cancer cases through linkage of the study participants to eight original and two additional (Arizona and Texas) state cancer registry databases. Cancer registries have been certified by the North American Association of Central Cancer Registries to capture at least 90% of cancer incidences within 2 years of cancer occurrence. Our case ascertainment method has been described previously, which demonstrated that approximately 90% of cancers were identified through cancer registries (Michaud *et al*, 2005). Cancer registry data included cancer diagnosed, diagnosis date, morphology code, grade, and stage information.

We defined cases as invasive, first primary epithelial ovarian cancer using the *International Classification of Diseases for Oncology, Third Edition* [16], code C56.9. Borderline and non-epithelial ovarian cancer cases were not included as cases in this study. We further classified the epithelial ovarian cancer into the following subtypes: serous (morphology code: 8260, 8441, 8450, 8460, 8461, 8462), endometrioid (morphology code: 8380, 8381, 8560, 8570), mucinous (morphology code: 8470, 8471, 8472, 8480, 8481, 8490), clear cell (morphology code: 8310, 8313), and other tumours.

### Statistical analysis

We estimated RRs and 95% CIs with Cox proportional hazards models (Cox, 1972) using the PHREG procedure in SAS (SAS, Institute , Cary, NC, USA). All reported *P*-values were two-sided. We calculated person-years of follow-up time from the date when the questionnaire was returned until participants were diagnosed with cancer, moved out of the cancer registry ascertainment area, died, or the end of follow-up was reached (31 December 2006), whichever occurred first. We confirmed the proportional hazards assumption was met for the main exposure and other covariates by including interaction terms with time and using the Wald *χ*^2^-procedure to test whether all coefficients equaled 0 (Ng’andu, 1997).

To adjust total energy intake in models, we used a nutrient density method, in which fat intake was expressed as a percentage of total energy intake. In analysis of sources and types of fat, we mutually adjusted for all other sources and types of fat. For example, the RR for saturated fat intake represents the association of ovarian cancer risk with the substitution of non-fat energy sources and is independent of monounsaturated and polyunsaturated fat intake (Willett, 1998).

We performed analyses using fat as either a categorical or continuous variable. We created quintiles and deciles of fat intake, based on the distribution in the study population. In categorical analyses, we also tested whether there was a linear trend across the categories using the median value of each category as a single continuous variable in a model. Before conducting analyses of fat intakes as continuous variables, we evaluated the relation of fat intake to ovarian cancer using a nonparametric regression curve that used a restricted cubic spline. We found the associations between total fat and all types of fat and ovarian cancer, including subtypes, were linear. Relative risks in continuous analyses were estimated for a 10% increase in total fat intake as percentage of total energy intake, and for a 5% increase in sources and types of fat intake as a percentage of total energy intake.

In multivariate models, we adjusted for age, race (white, non-Hispanic, black, non-Hispanic, others), education (<12 years, high-school graduate, some college, and college graduate/post college), BMI (<25, 25–30, 30+ kg m^−2^), family history of ovarian cancer (yes, no), duration of oral contraceptive use (0, 1–4, 5–9, 10+ years), parity (0, 1, 2, 3–4, and 5+ children), duration of MHT use (0, >0–<5, 5–9, 10+ years), and total energy intake. We created an indicator variable for missing responses in each covariate. The proportion of missing for each variable was generally <4%. In additional analyses, we further adjusted for physical activity and smoking and found that the results did not change. We also performed sensitivity analyses by excluding cases diagnosed within the first 2 years of follow-up and found similar results. To examine whether the associations for fat intake differed by oral contraceptive use, parity, MHT use, and BMI, we included cross product terms of the ordinal score of the level of each factor and fat intake expressed as a continuous variable in the multivariate models.

We conducted measurement error corrections for the association between total fat intake and risk of ovarian cancer using the calibration substudy data. Using a regression calibration method for the case of multiple covariates measured with error (Rosner *et al*, 1990; Spiegelman *et al*, 2001) and a SAS macro (Logan and Spiegelman, 2004), we simultaneously corrected for measurement error in fat and total energy intakes and adjusted for age, race, education, BMI, family history of ovarian cancer, oral contraceptive use, parity, and MHT use.

## Results

During an average of 9 years of follow-up, a total of 695 ovarian cancer cases were identified. Overall, 404 ovarian cancer cases were classified as serous tumour, 66 were endometroid, 36 were mucinous, 24 were clear cell tumours, and 165 were other epithelial tumour types or were not otherwise specified. Total fat intake ranged from 20% of total energy intake (10th percentile) to 40% of total energy intake (90th percentile). Median intake of fat from animal and vegetable sources was 14.1% and 11.7% of total energy intake, respectively. The major type of fat was monounsaturated fat (10th–90th percentile: 7–15%) followed by saturated fat (10th–90th percentile: 6–13%), and polyunsaturated fat (10th–90th percentile: 4–10%). These types of fat were correlated with each other (Pearson correlation coefficient between saturated fat and monounsaturated fat=0.82; between saturated and polyunsaturated=0.45; between monounsaturated and polyunsaturated=0.75). Major food sources of saturated fat were dairy (22%), meat (20%), and butter/margarine (16%). Foods contributing to monounsaturated fat intake were meat (21%), butter/margarine (12%), and vegetable oils including salad dressing (9%). Main sources of polyunsaturated fat intake were vegetable oils including salad dressing (20%), butter/margarine (13%), mayonnaise (11%), and meat (10%). In general, women in the highest quintile of total fat intake were more likely to be Caucasian, have a higher BMI, a lower educational level, higher parity, and have never used MHT (Table 1). The duration of oral contraceptive use did not differ by total fat intake.

We found that total fat intake was significantly positively associated with risk of ovarian cancer. Compared with the lowest quintile of total fat intake, women in the highest quintile had a 28% increased risk of ovarian cancer (multivariate RR_Q5 *vs* Q1_=1.28, 95% CI: 1.01–1.63, *P*_trend_=0.04; Table 2). When we examined the association by comparing the highest decile of total fat intake (median=43% of total energy from fat) with the lowest (median=17% of total energy from fat), we found a multivariate RR of 1.49 (95% CI: 1.06–2.11, *P*_trend_=0.03) for ovarian cancer. Fat intake from animal sources was significantly positively related to the risk of ovarian cancer (multivariate RR_Q5 *vs* Q1_=1.30, 95% CI: 1.02–1.66, *P*_trend_=0.03), but fat intake from plant sources was not (multivariate RR_Q5 *vs* Q1_=1.00, 95% CI: 0.79–1.27, *P*_trend_=0.96). We further examined whether types of fat were related to ovarian cancer risk. We observed that saturated and monounsaturated fat intakes were not related to risk of ovarian cancer, but polyunsaturated fat intake suggested a weak positive association with risk of ovarian cancer (multivariate RR_Q5 *vs* Q1_=1.28, 95% CI: 0.92–1.77, *P*_trend_=0.09). Intake of trans fat also showed a statistically nonsignificant positive association with risk of ovarian cancer (multivariate RR_Q5 *vs* Q1_=1.19, 95% CI: 0.94–1.50, *P*_trend_=0.15). When we excluded cases diagnosed during the first 2 years of follow-up to exclude cases that may have changed their diet due to early symptoms of cancer, we found that the results did not change: the multivariate RR_Q5 *vs* Q1_ were 1.34 (95% CI: 1.03–1.76) for total fat intake; 1.30 (95% CI: 1.00–1.70) for fat from animal food; 1.08 (95% CI: 0.82–1.41) for fat from plant food; 1.00 (95% CI: 0.66–1.52) for saturated fat intake; 1.01 (95% CI: 0.61–1.69) for monounsaturated fat; and 1.37 (95% CI: 0.95–1.97) for polyunsaturated fat.

In analyses using total fat intake as a continuous variable (Table 3), we observed an 11% increased risk of ovarian cancer per increment of 10% of total energy from fat (multivariate RR=1.11, 95% CI: 1.01–1.23) and 28% increased risk of ovarian cancer for polyunsaturated fat intake (multivariate RR=1.28, 95% CI: 1.02–1.61, per increment of 5% of total energy from polyunsaturated fat intake). When we corrected for measurement errors in the assessments of total fat intake, we found that the association between total fat intake and ovarian cancer became robust. After correction for measurement errors, the multivariate RR for ovarian cancer was 1.26 (95% CI: 1.02–1.55) for an increment of 10% of total energy from fat.

We further examined total fat intake in relation to ovarian cancer subtypes (Table 3). Intake of total fat as well as fat from animal sources was positively associated with risk of serous tumour, the most common histological subtype of ovarian cancer. The multivariate RR of serous tumour was 1.14 (95% CI: 1.00–1.30) for 10% increment of total energy from total fat and 1.10 (95% CI: 1.01–1.20) for 5% increment of total energy from fat from animal sources. Fat intake was not related to risk of endometroid tumour, whereas results for mucinous tumour were similar to those of serous tumour. However, due to low numbers for endometroid and mucinous tumour, the power to detect an association may have been insufficient. There were no statistically significant heterogeneities in associations of fat intake and ovarian cancer subtypes (*P*-tests for heterogeneities >0.17).

**Figure 1:**
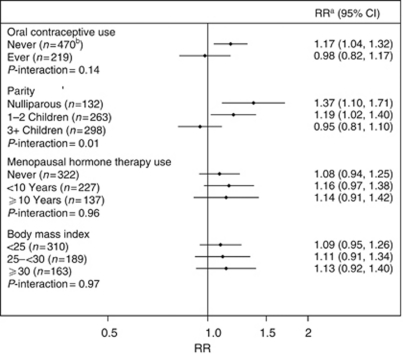
Multivariate RRs (adjusted for age, race (white, non-Hispanic, black, non-Hispanic, others), education (<12 years, high-school graduate, some college, and college graduate/post college), BMI (<25, 25–30, 30+ kg m^−2^), family history of ovarian cancer (yes, no), duration of oral contraceptive use (0, 1–4, 5–9, 10+ years), parity (0, 1, 2, 3–4, and 5+ children), duration of MHT use (0, >0 to <5, 5–9, 10+ years), and total energy intake (continuous)) and 95% CIs of ovarian cancer for total fat intake by oral contraceptive use, parity, MHT use, and BMI in the NIH-AARP Diet and Health Study, continuous model. ^a^Increment of 10% of total energy from fat. ^b^Number of cases.

We found a statistically significant interaction for the association between total fat intake and risk of ovarian cancer by parity (*P*-value for interaction=0.01); however, there were no interactions by oral contraceptive use, MHT use, or BMI (Figure 1). The association between total fat intake and risk of ovarian cancer was significant in women who were nulliparous (multivariate RR=1.37, 95% CI: 1.10–1.71 for increment of 10% of total energy from fat) and in women who had 1–2 children (multivariate RR=1.19, 95% CI: 1.02–1.40), but not in women who had more than three children (*P*-value for interaction=0.01). Although no significant interaction by oral contraceptive use was observed, the association between total fat intake and ovarian cancer was stronger among women who never took oral contraceptives (multivariate RR=1.17, 95% CI: 1.04–1.32 for increment of 10% of total energy intake) than among women who had ever taken oral contraceptives (multivariate RR=0.98, 95% CI: 0.82–1.17). Menopausal hormone therapy use and BMI did not modify the association between total fat intake and risk of ovarian cancer.

## Discussion

In this large prospective cohort study, we found that intakes of total fat and fat from animal sources were significantly positively associated with risk of ovarian cancer. Intakes of saturated and monounsaturated fat were not related to risk of ovarian cancer, whereas polyunsaturated fat intake showed a weak positive association. Serous tumour, the most common histological subtype of ovarian cancer, showed similar results as total ovarian cancer. The association between total fat intake and risk of ovarian cancer was stronger in subgroups of women who were nulliparous or never used oral contraceptives.

In contrast to findings from a pooled analysis of 12 prospective cohort studies and recent studies, we found a positive association between total fat intake and risk of ovarian cancer. The California Teachers Study, which was not included in the pooled analysis, found no association with the total fat intake (RR_Q5 *vs* Q1_=0.85, 95% CI: 0.58–1.24, 280 cases) (Chang *et al*, 2007). Recently, the Netherlands Cohort study also reported no association of total fat with risk of ovarian cancer (Gilsing *et al*, 2011). Most cohort studies that examined fat intake in relation to ovarian cancer tended to a narrow range of total fat intake. This limited the ability to examine more extreme contrasts of total fat intake, thus associations may not have been detected, if they exist. On the other hand, the distribution of total fat intake in our study showed a wide range: the top 10 percentile of our study participants had total fat intake more than 40% of total energy from fat, whereas the bottom 10 percentile of participants had less than 20% of total energy from fat. The wide range of fat intake in our study may be, in part, due to measurement error inherent to the food frequency questionnaire. Nevertheless, measurement error in the food frequency questionnaire used in our study is comparable to that in the food frequency questionnaire used in other cohort studies (Subar *et al*, 2001). In addition, we corrected for measurement errors in total fat intake and found that the positive association became stronger, but did not appreciably change. Our finding is also supported by the Women's Health Initiative Dietary Modification Randomized Controlled Trial that investigated the effect of a low-fat dietary pattern in postmenopausal women (Prentice *et al*, 2007). Mean fat intake in the intervention and the control group at year 1 after randomisation was 24% and 35% of total energy intake from fat, respectively. After 4–8 years of follow-up, this trial found that ovarian cancer risk was significantly lower in the intervention group (hazard ratio=0.60, 95% CI: 0.38–0.96) than in the control group.

We observed that fat intake from animal sources, but not from plant sources, was significantly positively associated with risk of ovarian cancer. This is consistent with findings from other cohort studies: a pooled analysis aforementioned reported a suggestive increase in risk of ovarian cancer for fat intake from animal sources (RR_Q1 *vs* Q4_=1.15, 95% CI: 0.99–1.33, *P*_trend_=0.15) and the Netherlands Cohort study also observed an increased risk of ovarian cancer with increased intake of fat from animal foods (RR=1.11, 95% CI: 0.99–1.25 for 13 g per day increment). In addition, a case–control study that examined dietary changes over time observed that substituting non-animal fat for animal fat between 2 and 7 years before cancer diagnosis or study recruitment was related to 35% lowered risk of ovarian cancer (odds ratio=0.65, 95% CI: 0.50–0.85, for 100 kcal increment) (Lubin *et al*, 2006). In examining types of fat in relation to ovarian cancer, both a meta-analysis of case–control studies and the pooled analysis of cohort studies observed a positive association with saturated fat intake. However, our study found no association of saturated fat with risk of ovarian cancer.

Some studies suggested that reproductive and hormonal risk factors for ovarian cancer differ by histological types (Tung *et al*, 2003; Soegaard *et al*, 2007; Gates *et al*, 2010). However, ours and two other studies (Risch *et al*, 1996; Genkinger *et al*, 2006) that examined the association between fat intake and ovarian cancer by histological type did not observe a significant difference in risk estimates by histological type. Owing to the small number of cases in mucinous, endometrioid, and clear cell tumours, studies had limited ability to examine these associations. Further examination of these relationships in histological subtypes is warranted.

Unlike a pooled analysis that found no effect modification by reproductive and hormonal factors (Genkinger *et al*, 2006), we found that the association between total fat intake and ovarian cancer risk was modified by parity. A significantly increased risk of ovarian cancer for total fat intake was observed in women with no child or 1–2 children, but not in women with 3+ children. In addition, although the interaction was not statistically significant, the positive association between total fat and risk of ovarian cancer was significant in women who never used oral contraceptives, but not in women ever used oral contraceptives. Given that oral contraceptive use and parity have been identified as protective factors for ovarian cancer, women who did not use oral contraceptives or who were nulliparous may be at a higher risk of ovarian cancer and more susceptible to the detrimental effect of fat on ovarian cancer risk. On the other hand, among oral contraceptive users and parous women, the protective effect of oral contraceptive use and pregnancy may override the harmful effect of high fat intake. Interestingly, the Netherlands Cohort Study also observed that the relation of animal fat intake to ovarian cancer risk was stronger in women who never used oral contraceptive use. In contrast to our findings, this study observed a significant association between animal fat intake and risk of ovarian cancer in parous women, but not in nulliparous women. These findings also warrant further investigation.

Our study has several limitations. Diet was assessed only once at baseline, as opposed to repeated assessments during follow-up, therefore we were not able to examine the changes in fat intake over time. Similarly, we did not assess fat intake at earlier stages of life, particularly during reproductive years that may be relevant to lifelong risk. Measurement error is an inherent limitation in self-reported dietary assessment. Nevertheless, we were able to correct measurement error using the calibration study data. Despite a large number of ovarian cancer cases, we were not able to examine the association by specific subtypes due to the rarity of some histological types. The ovarian cancer subtypes were defined using pathology information provided by state cancer registries and we were not able to review them. Thus, some misclassification of subtypes may have occurred.

Our study also has several strengths. This is a large prospective cohort study in which diet was measure at baseline, thus decreasing the likelihood of recall bias. In addition, our study had wide ranges of dietary fat intake providing good statistical power. We were also able to examine subtypes of ovarian cancer and adjust for potential ovarian cancer risk factors in addition to performing stratified analyses by several risk factors.

In conclusion, our findings support the hypothesis that fat intake, particularly fat from animal sources, increases risk of ovarian cancer. The association of total fat intake with ovarian cancer may be modified by hormone and reproductive factors such as parity and oral contraceptive use. Further investigation is needed to better understand these associations.

## Figures and Tables

**Table 1:** Characteristics of women by quintiles of total fat intake in the NIH-AARP Diet and Health Study

	**Quintiles of total fat intake**				
	**1**	**2**	**3**	**4**	**5**
*N*	30 310	30 311	30 311	30 311	30 310
Age at study entry (years)^a^	61.8 (5.4)	61.8 (5.4)	61.7 (5.5)	61.7 (5.4)	61.6 (5.4)
Body mass index (kg m^−2^)^a^	25.4 (5.3)	26.3 (5.6)	26.9 (5.9)	27.3 (6.2)	27.9 (6.9)
Total energy intake (kcal)^a^	1465 (620)	1491 (594)	1555 (623)	1632 (663)	1707 (715)
White, non-Hispanic (%)	89	90	90	90	91
College and post-graduate (%)	38	35	32	28	24
Family history of ovarian cancer (%)^b^	3	4	4	4	4
					
*Oral contraceptives use (%)* c					
Never or <1 year	59	59	58	59	59
1–4 Years	18	18	18	18	18
5–9 Years	13	13	13	13	12
10+ Years	9	10	10	10	10
					
*Parity (%)* c					
0	17	16	15	15	14
1 Child	11	10	10	10	10
2 Children	28	27	26	25	25
3+ Children	45	48	48	50	51
					
*Menopausal hormone therapy use (%)* c					
Never	50	50	51	53	56
<10 Years	35	35	33	32	29
10+ Years	13	14	14	14	13

a Mean and standard deviation.

b From a subcohort.

c Do not add up to 100% due to missing category.

**Table 2:** Relative risks and 95% confidence intervals of ovarian cancer by quintiles of total fat intake in the NIH-AARP Diet and Health Study

	**Quintiles of total fat intake** a					
	**1**	**2**	**3**	**4**	**5**	***P*_trend_**
*Total fat*						
Median intake^b^	19.9	25.7	29.8	33.9	39.9	
Cases (*N*)/Person-years	126/285 549	126/285 533	159/284 164	130/281 952	154/280 665	
Age-adjusted	1.00	1.00 (0.78–1.28)	1.27 (1.01–1.61)	1.05 (0.82–1.34)	1.26 (0.99–1.59)	0.06
Multivariate^c^	1.00	1.00 (0.79–1.29)	1.29 (1.02–1.63)	1.06 (0.83–1.36)	1.28 (1.01–1.63)	0.04
						
*Fat from animal sources*						
Median intake^b^	7.9	11.3	14.1	17.1	22.0	
Cases (*N*)/Person-years	127/285 983	137/285 951	136/284 164	142/282 240	153/279 526	
Age-adjusted	1.00	1.09 (0.85–1.38)	1.09 (0.86–1.39)	1.16 (0.91–1.47)	1.27 (1.00–1.61)	0.04
Multivariate^c^	1.00	1.09 (0.86–1.39)	1.11 (0.87–1.41)	1.18 (0.92–1.50)	1.30 (1.02–1.66)	0.03
						
*Fat from plant sources*						
Median intake^b^	6.4	9.3	11.7	14.5	19.5	
Cases (*N*)/Person-years	133/283 730	129/284 373	160/284 157	138/283 276	135/282 627	
Age-adjusted	1.00	0.97 (0.76–1.24)	1.20 (0.95–1.51)	1.03 (0.81–1.31)	1.01 (0.79–1.28)	0.92
Multivariate^c^	1.00	0.96 (0.75–1.22)	1.18 (0.94–1.49)	1.02 (0.80–1.30)	1.00 (0.79–1.27)	0.96
						
*Saturated fat*						
Median intake^b^	5.7	7.5	9.0	10.6	13.2	
Cases (*N*)/Person-years	126/285 889	143/285 801	144/284 051	147/282 055	135/280 067	
Age-adjusted	1.00	1.14 (0.90–1.45)	1.16 (0.91–1.47)	1.20 (0.95–1.53)	1.11 (0.87–1.42)	0.39
Multivariate^c^	1.00	1.07 (0.80–1.42)	1.06 (0.77–1.46)	1.10 (0.78–1.55)	1.03 (0.71–1.50)	0.98
						
*Monounsaturated fat*						
Median intake^b^	7.1	9.4	11.1	12.7	15.1	
Cases (*N*)/Person-years	125/285 597	132/285 025	157/284 257	134/282 618	147/280 366	
Age-adjusted	1.00	1.06 (0.83–1.36)	1.27 (1.00–1.60)	1.09 (0.86–1.39)	1.21 (0.95–1.54)	0.13
Multivariate^c^	1.00	1.00 (0.73–1.37)	1.14 (0.79–1.64)	0.95 (0.63–1.43)	1.01 (0.63–1.60)	0.87
						
*Polyunsaturated fat*						
Median intake^b^	4.5	5.8	6.8	8.0	10.2	
Cases (*N*)/Person-years	125/283 971	125/284 901	144/283 464	139/283 363	162/282 165	
Age-adjusted	1.00	1.00 (0.78–1.28)	1.15 (0.91–1.47)	1.11 (0.87–1.41)	1.30 (1.03–1.64)	0.02
Multivariate^c^	1.00	0.97 (0.74–1.27)	1.11 (0.83–1.47)	1.08 (0.80–1.47)	1.28 (0.92–1.77)	0.09

a Quintiles based on the distribution in the study population. Cutpoints for each quintile are 23.2, 27.8, 31.8, and 36.4 for total fat; 9.8, 12.7, 15.5, and 19.1 for fat from animal sources; 8.0, 10.4, 13.0, and 16.5 for fat from plant sources; 6.7, 8.3, 9.8, and 11.6 for saturated fat; 8.4, 10.3, 11.9, and 13.7 for monounsaturated fat; 5.2, 6.3, 7.4, and 8.9 for polyunsaturated fat.

b Percent from total energy intake from fat.

c Adjusted for age, race (white, non-Hispanic, black, non-Hispanic, others), education (<12 years, high-school graduate, some college, and college graduate/post college), body mass index ( <25, 25–30, 30+ kg m^−2^), family history of ovarian cancer (yes, no), duration of oral contraceptive use (0, 1–4, 5–9, 10+ years), parity (0, 1, 2, 3–4, and 5+ children), duration of menopausal hormone therapy use (0, >0-<5, 5–9, 10+ years), and total energy intake (continuous).

d Mutually adjusted for all other sources and types of fat in analysis of sources and types of fat.

**Table 3:** Multivariate relative risks^a^ and 95% confidence intervals for histological subtypes of ovarian cancer in the NIH-AARP Diet and Health Study, continuous model

	**Increment (%)** b	**All ovarian cancer**	**Serous tumour**	**Endometroid tumour**	**Mucinous tumour**
Cases (*n*)		695	404	66	36
Total fat	10	1.11 (1.01–1.23)	1.14 (1.00–1.30)	0.91 (0.66–1.25)	1.42 (0.93–2.17)
Fat from animal sources	5	1.09 (1.02–1.17)	1.10 (1.01–1.20)	0.99 (0.80–1.24)	1.37 (1.06–1.77)
Fat from plant sources	5	1.02 (0.95–1.10)	1.02 (0.93–1.12)	0.93 (0.73–1.18)	1.02 (0.74–1.39)
Saturated fat	5	1.14 (0.93–1.40)	1.16 (0.89–1.52)	1.28 (0.63–2.62)	1.39 (0.61–3.14)
Monounsaturated fat	5	0.88 (0.67–1.14)	0.96 (0.68–1.36)	0.49 (0.20–1.21)	1.06 (0.36–3.15)
Polyunsaturated fat	5	1.28 (1.02–1.61)	1.13 (0.83–1.53)	1.78 (0.87–3.64)	1.20 (0.46–3.15)

a Adjusted for age, race (white, non-Hispanic, black, non-Hispanic, and others), education (<12 years, high-school graduate, some college, and college graduate/post college), body mass index (<25, 25–30, 30+ kg m^−2^), family history of ovarian cancer (yes, no), duration of oral contraceptive use (0, 1–4, 5–9, 10+ years), parity (0, 1, 2, 3–4, and 5+ children), duration of menopausal hormone therapy use (0, >0-<5, 5–9, 10+ years), and total energy intake (continuous).

b Percent of total energy from fat.
